# Parental satisfaction of U.S. physicians: associated factors and comparison with the general U.S. working population

**DOI:** 10.1186/s12909-016-0737-7

**Published:** 2016-08-27

**Authors:** Tait D. Shanafelt, Omar Hasan, Sharonne Hayes, Christine A. Sinsky, Daniel Satele, Jeff Sloan, Colin P. West, Lotte N. Dyrbye

**Affiliations:** 1 Mayo Clinic, Rochester, MN USA; 2 American Medical Association, Chicago, IL USA; 3 Department of Internal Medicine, 200 First Street, Rochester, MN 55905 USA; 4 Division of Biomedical Statistics and Informatics, Mayo Clinic, Rochester, MN USA

**Keywords:** Physicians, Doctors, Parents, Satisfaction, Children, Career, Work-life integration

## Abstract

**Background:**

Physicians work considerably longer hours and are less satisfied with work-life balance than U.S. workers in other fields. There is, however, minimal data on physicians’ parental satisfaction.

**Methods:**

To evaluate differences in parental satisfaction among physicians and workers in other fields, we surveyed U.S. physicians as well as a probability-based sample of the general U.S. working population between August 2014–October 2014. Parental satisfaction and the perceived impact of career on relationships with children were evaluated.

**Results:**

Among 6880 responding physicians (cooperation rate 19.2 %), 5582 (81.1 %) had children. Overall, physicians were satisfied in their relationships with their children, with 4782 (85.9 %) indicating that they were either very satisfied [*n* = 2738; (49.2 %)] or satisfied [*n* = 2044 (36.7 %)]. In contrast, less than half believed their career had made either a major [*n* = 1212; (21.8 %)] or minor positive [*n* = 1260; (22.7 %)] impact on their relationship with their children, with a slightly larger proportion indicating a major (*n* = 2071 [37.2 %]) or minor (*n* = 501 [9 %]) negative impact. Women physicians were less likely to believe their career had made a positive impact as were younger physicians. Hours worked/week inversely correlated with the belief that career had made a positive impact on relationships with children. Both men (OR: 2.75; *p* < 0.0001) and women (OR: 4.33; *p* < 0.0001) physicians were significantly more likely to report that their career had a negative impact on relationships with their children than the sex-matched U.S. working population.

**Conclusions:**

U.S. physicians report generally high satisfaction in their relationships with their children. Despite their high satisfaction, physicians have a more negative perception of the impact of their career on relationships with their children than U.S. workers in general.

**Electronic supplementary material:**

The online version of this article (doi:10.1186/s12909-016-0737-7) contains supplementary material, which is available to authorized users.

## Background

Being a physician is a demanding and rewarding endeavor. Physicians undergo long and arduous training, work dramatically more hours than workers in other fields, and are at high risk for professional burnout [1]. As a counterbalance to these factors, physicians engage in intellectually stimulating work, are well compensated relative to many professions, and often develop meaningful and fulfilling relationships with patients [2, 3]. The net effect of these positive and negative forces on each physician is unique and can vary over the course of their career [4–6].

The impact of a career in medicine on physicians’ personal relationships has been a subject of interest in recent years. This dimension has taken on new complexity as the proportion of women physicians and two-physician couples have increased. Despite the stereotypes that physicians neglect their partners and have high divorce rates, recent studies in the U.S. suggest divorce rates among physicians are lower than both the population in general as well as other professionals [7]. Recent studies also suggest high marital satisfaction among spouses/partners of U.S. physicians and indicate that the amount of time spent together each day is the dominant driver of relationship satisfaction among spouses/partners of physicians [8].

While these studies have provided insights regarding the marital satisfaction of physicians, one aspect that has not been well studied is the impact of a career in medicine on physicians’ relationships with their children. The largest study exploring this dimension to date included only 415 physicians, excluded single parents, and was derived from a survey conducted >25 years ago [9]. That study found that physicians were less satisfied in their relationships with their children than their relationships with their spouse, with comparable parental satisfaction among both women and men physicians. On multivariate analysis, greater parental satisfaction was found among physicians less than age 45 and among those whose spouse was supportive of their career, as well as those married to an individual working as a professional or as a homemaker [9]. Physicians working in an employed practice model also had higher parental satisfaction than those working in solo or group practice.

A number of societal shifts have occurred since the time of that study, including an increase in the proportion of physicians who are women, a higher prevalence of single parents and a greater number of adults in two-career relationships [10]. Many changes have also occurred in the practice structure of medicine with a majority of physicians now functioning as employees of large medical organizations [11]. There are no data comparing the parental experience of physicians to that of the general U.S. population. Here we report the results of a large study evaluating the parental satisfaction of U.S. physicians along with comparison to the general U.S. population.

## Methods

As previously reported [12], we surveyed a national sample of U.S. physicians from all specialties between August 2014-October 2014 using the AMA’s Physician Master File, a nearly complete record of all physicians in the United States. Participation was voluntary and results were anonymous. In accordance with established methodology, the 35,922 physicians who opened at least one invitation e-mail were considered to have received the invitation to participate in the study [13]. A probability-based sample of employed U.S. adults was also surveyed for comparison to physicians in October 2014. The population survey was conducted using Knowledge Panel®, a probability-based panel designed to be representative of the U.S. population. Participants in the KnowledgePanel® are initially chosen scientifically by a random selection of telephone numbers and residential addresses. Persons in selected households are then invited by telephone or by mail to participate. Additional technical information is available at: http://www.gfk.com/products-a-z/us/knowledgepanelr-north-america/. The Mayo Clinic Institutional Review Board approved this study.

The initial results of this study, including the personal and professional characteristics of participating physicians, assessment of physician well-being (e.g. burnout, symptoms of depression, suicidal ideation, satisfaction with work/life balance, career satisfaction), and comparison of physician well-being to that of working U.S. adults have been previously reported [12].

### Parental satisfaction

Participating physicians were asked whether they had any children. Those who indicated they had children were asked the age of their youngest child and questions regarding their parental satisfaction (questions provided in Additional file 1). One item asked physicians to rate their overall satisfaction with the relationship they have with their children (response options: very satisfied, satisfied, neither satisfied nor dissatisfied, dissatisfied, very dissatisfied). The second item asked physicians to rate the impact their career had made on their relationship with their children (response options: major positive impact, minor positive impact, no impact, minor negative impact, major negative impact). These items were formatted to be similar to previously used items evaluating marital satisfaction [1, 8, 9]. The second question was also asked of working U.S. adults.

### Statistical analysis

Standard descriptive summary statistics were used to characterize physician and control samples. Associations between variables were evaluated using the Kruskal-Wallis test or Chi Square test as appropriate. All tests were two-sided with Type I error rates of 0.05. Multivariate analysis of differences across specialties was performed using logistic regression. Pooled multivariate logistic regression analysis of physicians with respect to the impact of their career on their relationship with their children was also performed to identify personal and professional factors associated with the dependent variable. For comparisons with population controls, physician data was restricted to responders between the ages of 29 and 65 years of age who were not retired in order to match the age and employment status of the U.S. population sample. All analyses were done using SAS version 9 (SAS Institute, Inc., Cary, North Carolina).

## Results

Of the 35,922 physicians who received an invitation to participate, 6880 (19.2 %) completed surveys [12]. Among the 6880 responding physicians, 5582 (81.1 %) indicated that they had children. The personal and professional characteristics of physicians with children, as well as all responding physicians, are shown in Table 1.

**Table 1:** Characteristics of physicians with children relative to all physician responders

Characteristics	2014 Responders *N* = 6880	Parents only *N* = 5582
Gender		
Male	4497(67.5 %)	3986 (71.9 %)
Female	2162(32.5 %)	1556 (28.1 %)
Missing	221	40
Age in Years		
Median	56	57
</= 35	466 (7.0 %)	244 (4.4 %)
36–45	1212 (18.2 %)	919 (16.6 %)
46–55	1484 (22.3 %)	1240 (22.4 %)
56–65	2206 (33.1 %)	1937 (35.0 %)
>65	1289 (19.4 %)	1201 (21.7 %)
missing	223	41
Relationship Status		
Missing	209	32
Single	722 (10.8 %)	303 (5.5 %)
Married	5573 (83.5 %)	5004 (90.2 %)
Partnered	274 (4.1 %)	156 (2.8 %)
Widowed	102 (1.5 %)	87 (1.6 %)
Age Youngest Child		
<5	740 (11.2 %)	740 (13.3 %)
5–12	873 (13.2 %)	873 (15.7 %)
13–18	781 (11.8 %)	781 (14.1 %)
19–22	737 (11.1 %)	737 (13.3 %)
22+	2420 (36.5 %)	2420 (43.6 %)
Missing	255	31
Hours Worked Per Week		
<40 h	1172(17.4 %)	990 (18.1 %)
40–49 h	1340(19.9 %)	1066 (19.5 %)
50–59 h	1667(24.7 %)	1350 (24.6 %)
60–69 h	1526(22.6 %)	1238 (22.6 %)
70–79 h	535(7.9 %)	436 (8.0 %)
≥80 h	509(7.5 %)	399 (7.3 %)
Missing	131	103
# Nights on Call Per Week		
Median(IQR)	1(0–3)	1 (0–3)
Primary Practice Setting		
Private practice	3605(52.6 %)	3048 (54.8 %)
Academic Medical Center	1625(23.7 %)	1251 (22.5 %)
Veterans hospital	104(1.5 %)	74 (1.3 %)
Active military practice	58(0.8 %)	37 (0.7 %)
Not in practice or retired	160(2.3 %)	136 (2.4 %)
Other	1303(19 %)	1020 (18.3 %)
Missing	205	16
Level of satisfaction relationship with children		
Very satisfied		2738 (49.2 %)
Satisfied		2044 (36.7 %)
Neither satisfied/dissatisfied		341 (6.1 %)
Dissatisfied		372 (6.7 %)
Very dissatisfied		73 (1.3 %)
No children/missing		14
Impact of career on relationship with children		
Major positive impact		1212 (21.8 %)
Minor positive impact		1260 (22.7 %)
No impact		517 (9.3 %)
Minor negative impact		2071 (37.2 %)
Major negative impact		501 (9.0 %)
No children/missing		21

### Parental satisfaction of U.S. physicians

Overall, physicians were satisfied in their relationships with their children, with 4782 (85.9 %) indicating that they were either very satisfied [*n* = 2738; (49.2 %)] or satisfied [*n* = 2044; (36.7 %)]. Slightly less than half of physicians believed their career had made either a major positive [*n* = 1212; (21.8 %)] or a minor positive [*n* = 1260; (22.7 %)] impact on their relationships with their children, while fewer believed their career had made a minor [*n* = 2071; (37.2 %)] or major negative [501; (9 %)] impact, respectively.

**Fig. 1:**
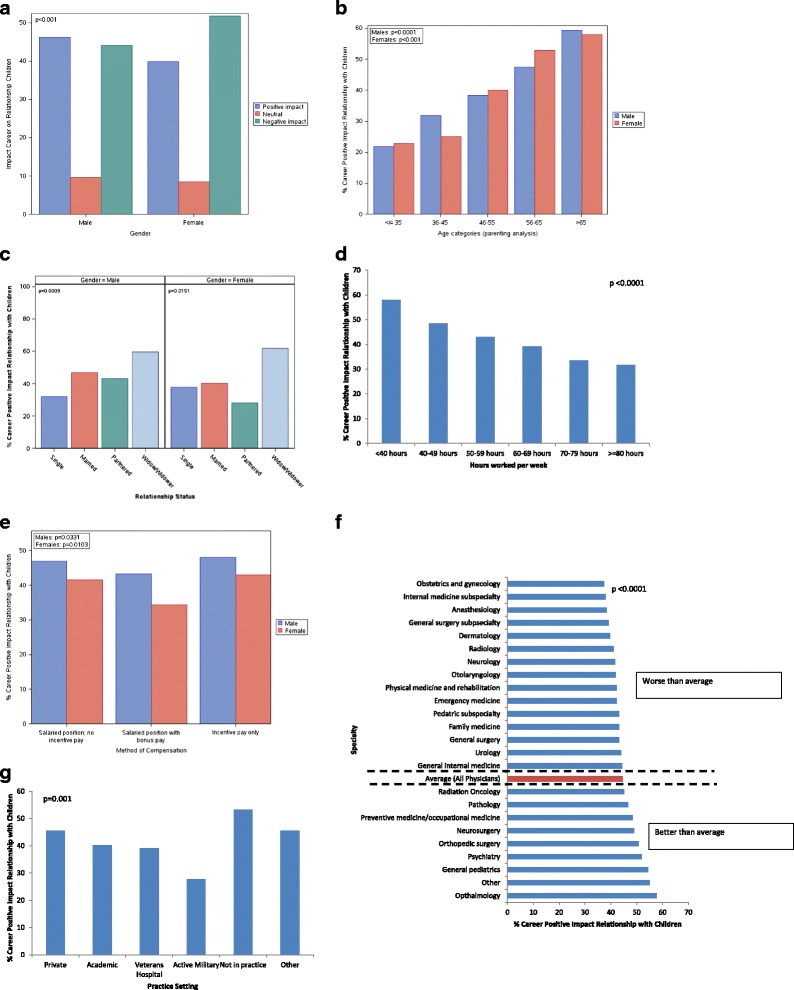
Physicians’ Perception of the Impact of Their Career On Relationships With Their Children. **a**. Impact of Career on Relationship with Children by Gender. **b**. Impact of Career on Relationship with Children by Age. **c**. Impact of Career on Relationship with Children by Relationship Status. **d**. Impact of Career on Relationship with Children by Hours worked/wk. **e**. Impact of Career on Relationship with Children by method of compensation. **f**. Impact of Career on Relationship with Children by Specialty. **g**. Impact of Career on Relationship with Children by practice setting

Small differences in satisfaction in relationships with their children were observed by gender, age, hours worked/week, nights on call/week, specialty, practice setting, or method of compensation (Additional file 1 Figure S1). Larger associations were observed between these personal and professional characteristics and physicians’ perception of the impact of their career on relationships with their children (Fig. 1a-f). Women physicians were more likely to report a negative impact of their career on relationships with their children than their male colleagues (women: 51.7 % vs. men: 41.1 %; p ˂ 0.001; Fig. 1a). Younger physicians were also less likely to believe their career had made a positive impact than their older colleagues (Fig. 1b). Physicians whose relationship status was single or partnered were less likely to be satisfied than those who were married (Fig. 1c). Hours worked/week inversely correlated with the belief that career had made a positive impact on relationships with their children, with an apparent dose effect (Fig. 1d). Those in a salary-plus-bonus model were less likely to believe their career had made a positive impact on relationships with their children than those in either a pure salaried position or a pure incentive-based system (Fig. 1e).

The association between specialty and practice setting and physicians’ belief that their career had made a positive impact on relationships with their children is shown in Fig. 1f and g. Specialties least likely to believe that their career had made a positive impact on relationships with their children included obstetrics and gynecology, internal medicine subspecialties, and anesthesiology, while those most likely to believe that their career had made a positive impact included ophthalmologists, general pediatricians, and psychiatrists.

The results of multivariate analysis including the variables age, gender, relationship status, hours/week, specialty, practice setting, and method of compensation are shown in Table 2. Women physicians and physicians who were older were more likely to be satisfied in their relationships with their children. Physicians whose relationship status was single or partnered were less likely to be satisfied than those who were married. Physicians who were in an academic or “other” practice setting were more likely to be satisfied than those in private practice, while those who were not in practice or retired were less likely to be satisfied. Physicians in a salaried model were less likely to be satisfied in their relationship with their children than those in a pure incentive-based compensation model. Hours worked/week also remained strongly associated with parental satisfaction in the multivariate analysis, where each additional hour worked/week reduced the likelihood of satisfaction by 1 %.

**Table 2:** Multivariate analysis to identify factors associated with satisfaction in relationship with children

Variable	OR (95 % CI)	*P*
Age (for each year older)	1.01 (1.00–1.02)	0.0070
Female (vs. Male)	1.29 (1.07–1.56)	0.0089
Partnered (vs. Married)	0.46 (0.31–0.68)	0.0001
Single (vs. Married)	0.60 (0.46–0.77)	<0.0001
Academic practice vs. private practice	1.50 (1.20–1.88)	0.0004
Not in practice/retired (vs. private practice)	0.54 (0.31–0.94)	0.0293
Other practice setting (vs. private practice)	1.31 (1.04–1.66)	0.0244
Salaried w no incentive pay (vs. incentive pay only)	0.72 (0.58–0.90)	0.0036
Hours worked per week (for each additional hour)	0.99 (0.98–0.99)	<0.0001

Factors in model: age, gender, relationship status, hours/week, specialty, practice setting, method of compensation

We also conducted a multivariate analysis including the same variables to identify factors associated with physicians’ perception that their career had made a positive impact on relationships with their children (Table 3). Increasing age (odds ratio [OR] = 1.039 [95 % confidence interval [CI]: 1.034-1.045]; p ˂ 0.0001) was associated with an increased likelihood of viewing career as a positive influence, while the number of hours worked/week (OR = 0.990 [95 % CI: 0.986-0.993]; p ˂ 0.0001) was inversely associated with the perception that career had made a positive impact. Physicians whose relationship status was single or partnered were less likely to view their career as a positive influence on relationship with children than those who were married. Physicians who specialized in general pediatrics and ophthalmology were more likely to believe that their career had made a positive impact than those in family medicine (used as the reference specialty).

**Table 3:** Multivariate analysis to identify factors associated with perception that career has had positive impact on relationship w/children in physicians

Variable	OR (95 % CI)	*P*
Age (for each year older)	1.039 (1.034–1.045)	<0.0001
Partnered (vs. Married)	0.693 (0.485–0.989)	0.0436
Single (vs. Married)	0.596 (0.461–0.769)	<0.0001
Hours worked per week (for each additional hour)	0.990 (0.986–0.993)	<0.0001
General Pediatrics as specialty (vs. Family Med)	1.657 (1.213–2.264)	0.0015
Ophthalmology as specialty (vs. Family Med)	1.432 (1.000–2.048)	0.0497

Factors in model: age, gender, relationship status, hours/week, specialty, practice setting, compensation

### Physicians compared to U.S. working population

**Fig. 2:**
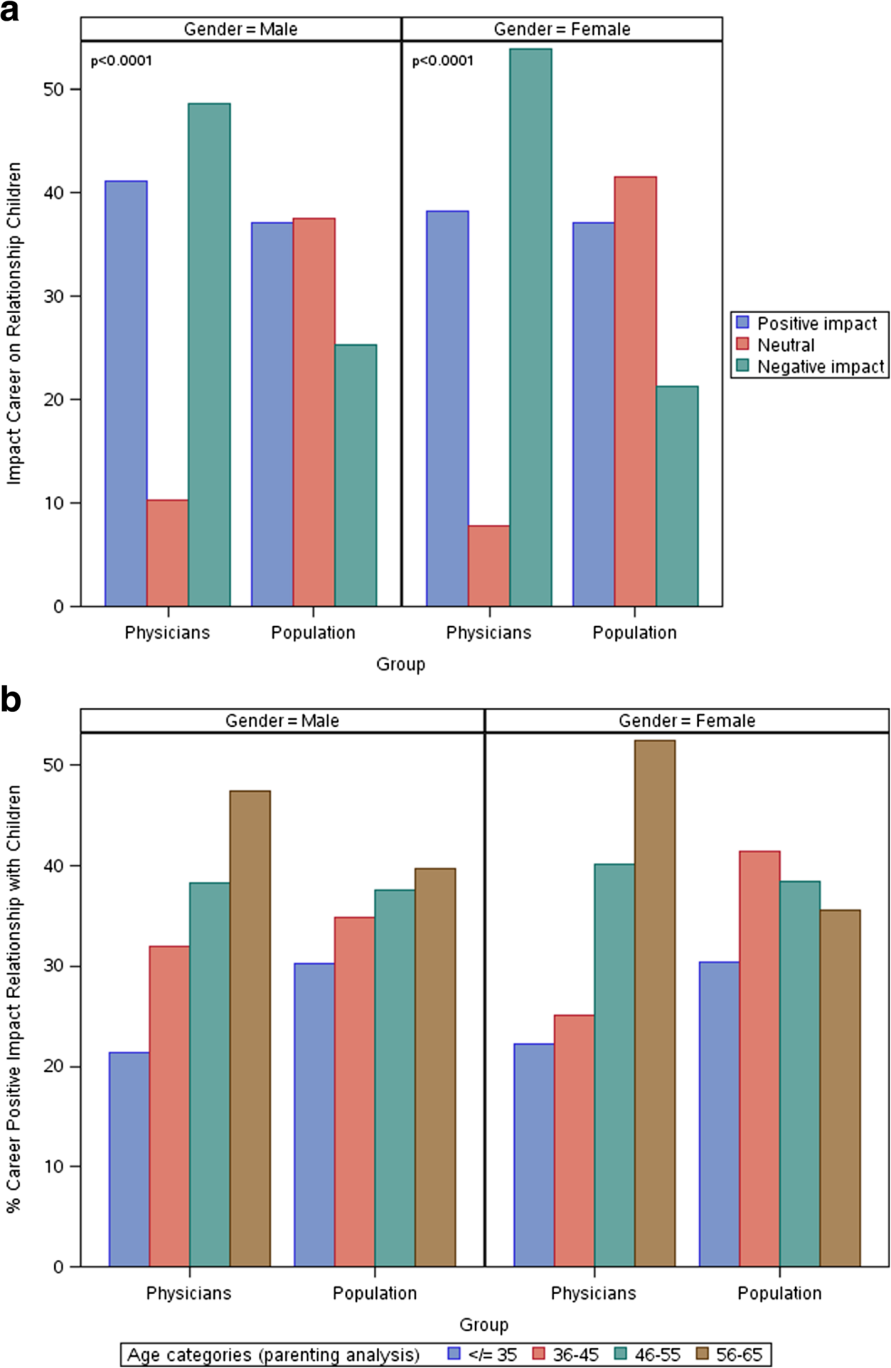
Perceived Impact of Career On Relationships With Their Children: Comparison of Physicians to U.S. Workers in General. **a** Impact of Career on Relationship with Children by Gender. **b** Impact of Career on Relationship with Children by Age

Next, we compared perceptions of the impact of career on relationships with children among physicians to the general U.S. working population (*n* = 3991; 74.2 %). Both men (physician 48.7 %; population 25.3 %; OR = 2.795 [95 % CI: 2.474-3.157]; p ˂ 0.001) and women (physician 54.0 %; population 21.3 %; OR = 4.327 [95 % CI: 3.715-5.041]; p ˂ 0.001) physicians were significantly more likely to report that their career had a negative impact on relationships with their children than U.S. workers (Fig. 2a). The strong relationship between increasing age and the perception that career had a positive impact on relationships with children was of greater magnitude among physicians than the general population for both men and women (Fig. 2b). Although the proportion of men and women physicians who believed that career had a positive impact on their relationships with children was lower for physicians than the age and sex-matched general population 45 and younger, it was similar to or higher than the age-matched general population for those age 46–65.

In the pooled (physicians and U.S. workers) multivariate analysis, the relationship between advancing age and a more favorable view of the impact of career on relationship with children persisted, as did the inverse association with hours worked/week. Single parents were less likely to view their career as a positive influence on relationship with children than those who were married. The effect of education on the perceived impact of career suggested a beneficial effect to higher levels of education. When compared to individuals who were high school graduates, a stepwise effect on likelihood of satisfaction was observed among individuals with an associate’s degree, bachelor’s degree, master’s degree, or professional/doctoral degree in a field other than medicine (i.e. greater likelihood of satisfaction with higher educational attainment). The beneficial effects of education also held true for physicians relative to high school graduates; however, the effect size of a doctoral degree in medicine degree appeared roughly equivalent to that of a bachelor’s degree, and was lower than that observed with a master’s degree or a professional/doctoral degree in a field outside medicine (Table 4).

**Table 4:** Predictors of career having positive impact on relationship with children in physicians and population norms

Variable	OR (95 % CI)	*P*
Age (for each year older)	1.026 (1.021–1.031)	<0.0001
Hours worked per week (for each additional hour)	0.986 (0.983–0.989)	<0.001
Single (vs. Married)	0.702 (0.601–0.820)	<0.0001
Some college, no degree (vs. High School Graduate)	1.394 (1.131–1.717)	0.0018
Associates Degree (vs. High School Graduate)	1.581 (1.247–2.004)	0.0002
Bachelors Degree (vs. High School Graduate)	2.144 (1.759–2.613)	<0.0001
Masters Degree (vs. High School Graduate)	2.570 (2.040–3.238)	<0.0001
Professional/Doctorate Degree (vs. High School Graduate)	2.868 (2.068–3.976)	<0.0001
Physicians (vs. High School Graduate)	2.117 (1.787–2.508)	<0.0001

Factors in model: age, gender, hours/week, relationship status, highest level of education

## Discussion

There is minimal information available regarding physicians’ parental satisfaction and perception of the impact of their career on their children. To our knowledge, this is the first national study to assess physicians’ parental satisfaction in the last 25 years, and the only study to evaluate a comparison cohort of working adults from the general U.S. population. Generally, physicians reported high levels of parental satisfaction. Despite the high satisfaction with their relationships with their children, nearly half of physicians reported their career had adversely impacted those relationships. Not surprisingly, hours worked/week had an inverse relationship with the likelihood of perceiving that career had a positive impact. Although women physicians were more likely to report a negative impact of career on relationship with children in univariate analysis, this difference did not persist on multivariate analysis adjusting for age and other factors. Collectively, these results suggest that, while physicians view their career as a potential barrier to the relationships they desire to have with their children, most are satisfied with relationships they develop nonetheless.

Both men and women physicians were dramatically more likely than U.S. workers in general to believe their career had a negative impact on their relationship with their children. Physicians also had a more polarized view of the impact of their career than U.S. workers; <10 % of physicians reported their career had “no impact” on their relationship with their children as compared to 35 %-40 % of U.S. workers. Notably, in multivariate analysis of both physicians and U.S. workers, advancing age was associated with a higher likelihood of viewing career as having a positive impact, while the number of hours worked/week had an inverse association with the perceived impact of career on relationships with children. We are unable to determine the potential reason for this association with age given the cross-sectional nature of the study. A variety of factors may contribute to the more favorable view of the impact of career on parenting with age including: i) a generational effect, ii) a change in the perceived impact of career for those having children later, iii) a change in perception of the impact of career on parenting as the parent and children get older, iv) differences in role conflict over the course of a career, and v) other unmeasured interacting variables factors (e.g. whether the individual is in a two career relationship and the professional characteristics of their partner).

In both physicians and workers in other fields, the highest level of education achieved was also related to the perceived impact of career on relationships with children independent of gender. Relative to high school graduates, a stepwise greater likelihood of reporting that career had a positive impact was observed for those with an associate’s degree, bachelor’s degree, master’s degree, and professional/doctoral degree in a field other than medicine. In contrast to this clear stepwise, incremental association between level of education and the perception that career had made a positive impact among non-physicians, the magnitude of effect of doctoral degree in medicine was more similar to that of a bachelor’s degree and lower than a master’s or professional/doctoral degree in other fields.

Collectively, these findings regarding the relationship between work hours, age and level of education with parental satisfaction are thought provoking, particularly for women. They may argue against the notion that pursuing an advanced or professional degree has an adverse impact on relationship with children and may also suggest that the perceived impact of career on relationships with children becomes more favorable with age. The multivariate analyses of physicians also provide some potential insights for physicians attempting to mitigate a negative effect of their career on their relationships with their children and seeking to cultivate parental satisfaction. First, they suggest that these individuals be acutely aware of the total number of hours worked more so than other variables. Second, for the most part, specialty is irrelevant with respect to satisfaction in relationship with children. Third, practice setting does appear to be related to satisfaction in relationship with children (higher for those in academic practice settings than private practice) but not impact of career on relationship with children. At a minimum, it would seem physician parents need to rigorously monitor work hours, periodically evaluate if they are spending too much time at work, and make sure their specialty and practice setting allow them adequate flexibility to tailor work hours to meet both personal and professional priorities. In this regard, it should be noted that reducing professional work hours may be a helpful strategy for many physicians [14–17], and that the available evidence suggests that working part time does not adversely impact quality of care [8, 9] or patient satisfaction [15, 18–21].

How do these results relate to previous reports? In 1988, Warde and colleagues surveyed 656 married physicians with children in southern California (single parents excluded) [9]. Parental satisfaction among physicians was somewhat lower than marital satisfaction in this cohort. Lower parental satisfaction was observed among older physicians and those experiencing greater “role conflict”, defined as “frustration with the competing demands of career, marriage, and family”. A second report from this cohort indicated greater role conflict was present among women physicians and younger physicians [22]. Among physicians younger than 45, 51 % of men and 87 % of women reported they had made career changes for their children. The most common career changes reported were decreasing work hours, a change in practice type, and interrupting career for their children [22]. More recent studies have also found work-home conflicts and how they are resolved to be powerful contributors to physician burnout [23–25].

Childrearing can be particularly challenging for women physicians due to cultural and societal parenting expectations [26–28]. In 1988, Levinson and colleagues reported the results of a seminal study of 862 women physicians working as faculty members of academic internal medicine practices who were under the age of 50 [29]. They found women physicians were more likely to delay childbearing compared to U.S. women and took relatively short maternity leaves [29]. Collectively, 87 % of academic women physicians reported that they relied on a paid employee to assist in the care of their children, and 68 % reported childrearing had slowed their career progress [29]. At the time of this study, few institutions provided job-sharing opportunities for these faculty members. Although a variety of initiatives to address some of these challenges specific to women physicians have been reported or proposed in recent years [30–33], much more progress is needed given the rapidly growing number of women physicians.

Our study is subject to a number of limitations. First, approximately one in five of physicians surveyed responded. As previously reported, the demographic characteristics of responders were generally consistent with U.S. physicians overall [12]. Nonetheless, how representative participants are of all physicians is unknown. Second, our data are cross-sectional in nature, and we are unable to assess longitudinal changes over time. Third, a number of variables that may interact with parental satisfaction (e.g. characteristics of partner and their career, marital discord, access to family support, access and affordability of day care) were not assessed. Fourth, the study describes the experience of physicians in the U.S. and its generalizability of physicians in other countries is unknown. Finally, although they are similar to previous questions evaluating parental satisfaction in other studies of physicians, the questions we used to evaluate parental satisfaction are not standardized instruments. The fact that we collected simultaneous information using one of these items in a sample of the general U.S. working population for comparison may, to some extent, mitigate this limitation.

Our study also has a number of important strengths. Participants in this study represent a large and diverse sample of U.S. physicians from all specialties and practice types. Unlike previous studies [9, 29], we did not limit the sample to physicians of a single gender, specialty, or practice setting, and we did not exclude single parents. The comparison of physicians to the general U.S. working population is also a unique contribution of this study.

## Conclusions

U.S. physicians report generally high satisfaction in their relationships with their children. Despite their high satisfaction, physicians have a more negative perception regarding the impact of their career on relationships with their children than other U.S. workers. The number of hours worked/week and specialty choice were strongly associated with the perceived impact of physicians’ career on their relationships with their children. Additional studies evaluating how physicians can promote healthy relationships with their children and the characteristics of the practice environment that facilitate healthy relationships would be useful.

## Additional file

Additional file 1: Survey Questions Related to Parental Satisfaction. **Figure S1.** Physicians’ Satisfaction with Relationship with Children. A. Satisfaction with Relationship with Children by Gender. B. Satisfaction with Relationship with Children by Age. C. Satisfaction with Relationship with Children by Relationship status. D. Satisfaction with Relationship with Children by Hours worked/wk. E. Satisfaction Relationship with Children by nights on call/week. F. Satisfaction Relationship with Children by Specialty. G. Satisfaction Relationship with Children by practice setting. H. Satisfaction Relationship with Children by method compensation. **Table S1.** Demographic Characteristics of Population Sample of Parents. (DOCX 73 kb)

## Abbreviations

CI: Confidence interval
OR: Odds ratio
US: United States
