# Supplementary material for: Parental satisfaction of U.S. physicians: associated factors and comparison with the general U.S. working population
Source: BMC Med Educ. 2016 Aug 27;16(1):228. doi: 10.1186/s12909-016-0737-7 (PMC5002113; doi:10.1186/s12909-016-0737-7)
Supplement: Additional file 1: — Survey Questions Related to Parental Satisfaction. Figure S1. Physicians’ Satisfaction with Relationship with Children. A. Satisfaction with Relationship with Children by Gender. B. Satisfaction with Relationship with Children by Age. C. Satisfaction with Relationship with Children by Relationship status. D. Satisfaction with Relationship with Children by Hours worked/wk. E. Satisfaction Relationship with Children by nights on call/week. F. Satisfaction Relationship with Children by Specialty. G. Satisfaction Relationship with Children by practice setting. H. Satisfaction Relationship with Children by method compensation. Table S1. Demographic Characteristics of Population Sample of Parents. (DOCX 73 kb) [file 12909_2016_737_MOESM1_ESM.docx]

Supplemental Data

1. Survey Questions Related to Parental Satisfaction:

Do you have any children?

a. yes

b. no

Following 3 items only asked of those with children

What age is your youngest child?

a. <5

b. 5-12

c. 13-18

d. 19-22

e. >23

Rate the impact your career has had on your relationship with your children:

1. major positive impact
2. minor positive impact
3. no impact
4. minor negative impact
5. major negative impact

Overall, rate your satisfaction with the relationship you have with your children?

a. very satisfied

b. satisfied

c. neither satisfied nor dissatisfied

d. dissatisfied

e. very dissatisfied

Supplemental Figure

A. Satisfaction with Relationship with Children by **GENDER**


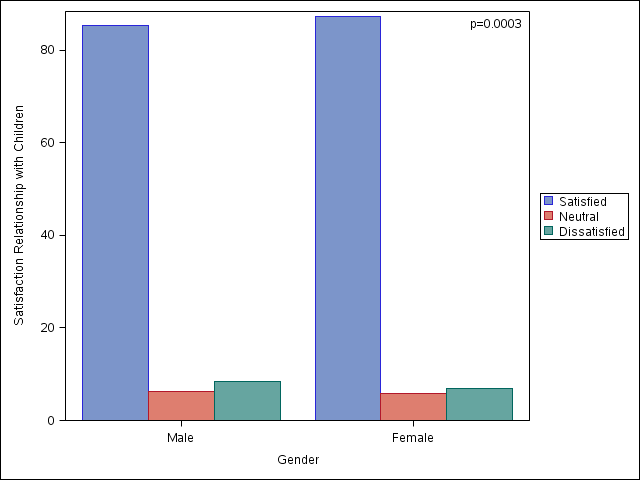


B. Satisfaction with Relationship with Children by **AGE**


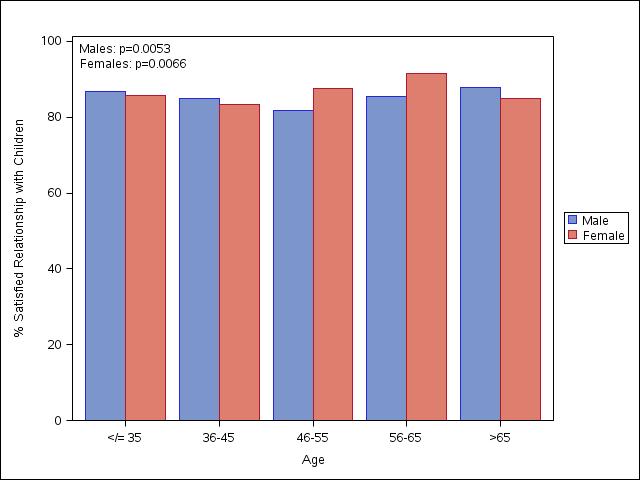


C. Satisfaction with Relationship with Children by **Relationship status**


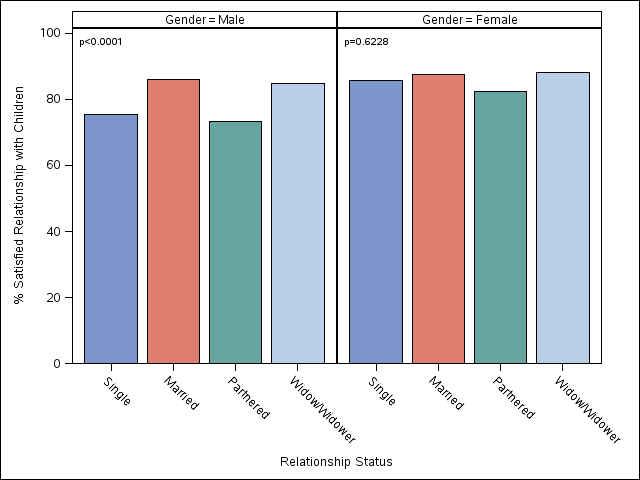


D. Satisfaction with Relationship with Children by **Hours worked/wk**

E. Satisfaction Relationship with Children by **nights on call/week**

F. Satisfaction Relationship with Children by **SPECIALTY**

G. Satisfaction Relationship with Children by **practice setting**

H. Satisfaction Relationship with Children by **method compensation**

Supplemental Table: Demographic Characteristics of Population Sample of Parents

**Note:** As discussed in the method section, physician data for this comparison was restricted to physicians between the ages of 29-65 who were not retired in order to match the age and employment status of the U.S. population sample

|  | Population  N=3991 |
| --- | --- |
| **Gender** |  |
| Male | 2152 (53.9%) |
| Female | 1839 (46.1%) |
| **Age** |  |
| Median | 53.0 |
|  |  |
| </=35 | 359 (9.0%) |
| 35-44 | 798 (20.0%) |
| 45-54 | 1327 (33.2%) |
| >55 | 1507 (37.8%) |
|  |  |
|  |  |
| **Relationship Status** |  |
| Single | 0 |
| Married | 643 (16.1%) |
| Partnered | 3092 (77.5%) |
| Widowed/widower | 182 (4.6%) |
| Missing | 74 (1.9%) |
|  |  |
| **Hours Worked/week** |  |
| Mean(SD) | 40.4 (11.4) |
| Median | 40.0 |
|  |  |
| <40 hrs | 1032 (25.9%) |
| 40-49 hrs | 2183 (54.7%) |
| 50-59 hrs | 522 (13.1%) |
| 60-69 hrs | 192 (4.8%) |
| 70-79 hrs | 27 (0.7%) |
| >80 hrs | 32 (0.8%) |
| Missing | 3 |
|  |  |
| **Highest Level of Education Completed** |  |
| Less than high school graduate | 146 (3.7%) |
| High school graduate | 906 (22.7%) |
| Some college, no degree | 810 (20.3%) |
| Associate degree | 495 (12.4%) |
| Bachelor’s degree | 942 (23.6%) |
| Master’s degree | 506 (12.7%) |
| Professional or Doctorate degree(other than MD/DO) | 186 (4.7%) |
| Missing | 0 |
| **Occupation** |  |
| Professional^a^ | 1754 (44.5%) |
| Health Care^b^ | 305 (7.7%) |
| Service^c^ | 267 (6.8%) |
| Sales^d^ | 306 (7.8%) |
| Office and Administrative Support | 306 (7.8%) |
| Farming, Forestry Fishing | 16 (0.4%) |
| Precision Production, Craft and Repair^e^ | 284 (7.2%) |
| Transportation and Material Moving | 113 (2.9%) |
| Armed services | 23 (0.6%) |
| Other | 568 (14.4%) |
| Missing | 49 |

^a^ business/financial, management, computer/mathematical, architecture/engineering, lawyer/judge, life/physical/social sciences, community/social services, teacher non-university, teacher college/university, other

^b^ nurse, pharmacist, paramedic, lab technician, nursing aide, orderly, dental assistant

^c^ protective service, food preparation/service, building cleaning/maintenance, personal care/service

^d^ sales representative, retails sales, other sales

^e^ construction and extraction, installation/maintenance/repair, precision production(machinist, welder, backer, printer, tailor)
